# Beyond a shared experience: Queer and trans youth navigating COVID-19

**DOI:** 10.1177/1473325020973329

**Published:** 2021-03

**Authors:** Megan S Paceley, Sloan Okrey-Anderson, Jessica N Fish, Lauren McInroy, Malcolm Lin

**Affiliations:** School of Social Welfare, University of Kansas, Lawrence, USA; College of Education and Human Development, University of Minnesota, St. Paul, USA; School of Public Health, University of Maryland, College Park, USA; College of Social Work, The Ohio State University, Columbus, USA; School of Social Welfare, University of Kansas, Lawrence, USA

**Keywords:** LGBTQ, social work practice, reflexivity

## Abstract

As queer scholars engaged in research and practice with queer and trans youth across the United States and internationally, we are deeply concerned about the impact of the COVID-19 pandemic on this vulnerable population. Physical distancing, social isolation, and school closures create challenges for adolescents as they navigate increasing independence from caregivers and more intimate relationships with peers. The challenges related to the ongoing pandemic are exacerbated for queer and trans youth as they negotiate their sexuality and gender identit(ies) in addition to typical developmental activities. Simultaneously, social work practices that provide critical and lifesaving support for queer and trans youth have been hindered by the closures and other pandemic-related changes to schools and community programs. We present this reflexive essay to deconstruct the notion of a shared experience during this pandemic and elevate the voices of queer and trans youth during this unprecedented moment in time. Through engagement with a small, local group of queer and trans youth, we share the challenges they are facing–particularly the consequences of social isolation and lack of identity-affirming support caused by physical distancing measures. We end by sharing their ideas for social work practice and how they can best be supported during this time. We do this to emphasize variations in the shared experience of a global pandemic and ensure the experiences of queer and trans youth are documented during this moment in history.

A global pandemic is in many ways a shared experience. As scholars and educators, we navigated this new reality by rapidly transitioning our classes online and adapting to all-day zoom meetings. As queer scholars, social workers, and family practitioners, however, we contend that the shared experience ends where marginalization begins. Our work with queer and trans youth sheds light on the very real challenges and disparities these youth are experiencing because of the ongoing crisis. Concurrently, physical distancing measures, school closures, and other pandemic-related changes have inhibited social work practice opportunities that provide critical and lifesaving support for queer and trans youth.

In this reflexive essay, we center queer and trans youth’s voices and expertise regarding the ways in which they are uniquely impacted by the isolation and lack of supports caused by physical distancing measures. We began with critical conversations with a small group of queer and trans youth in one community, which provided a local and contextualized picture of the realities of COVID-19 for this population. With their permission, we share their experiences, challenges, and concerns, as well as suggestions for social work practitioners and other helping professionals. We write this essay with the goal of documenting the lived experiences of queer and trans youth during COVID-19 and to call attention to the ways in which social work practitioners can acknowledge and address these challenges.

Before continuing, we must note our positionalities and privileges. As queer scholars, we have our own lived experiences as queer and/or trans individuals, yet we are adults during the COVID-19 pandemic. We are social work and family science scholars with practice and research experience with queer and trans youth, yet we recognize the power and status we hold as academics writing *about* queer and trans youth. We approach this essay from the stance that queer and trans youth are the experts on their own experiences and needs; we aim to center them as such.

## The pride alliance

The Pride Alliance is a leadership board comprised of six 15-19 year-old queer and trans youth. They started meeting in January 2020–two months before the COVID-19 pandemic began impacting their lives. For those two months, they met bi-weekly in-person and shared their expertise on queer youth research, providing incredible insight and recommendations. They also collectively organized and planned for local resources to support queer and trans youth. By March 2020 they were in the midst of planning a large event with the goal of building community and reducing isolation. They were collaborating closely and taking an active role in improving research and their community to better support queer and trans youth.

When COVID-19 arrived in the U.S., youth were suddenly homebound as schools transitioned to online instruction and state-sanctioned physical distancing orders were implemented. The Pride Alliance’s community event was canceled and their meetings were moved to video chats. The first few online meetings were awkward but productive. The youth continued to attend and were actively engaged, yet they were different than before–less excited, more somber. In April, we discussed the possibility of this essay with them, asking if they thought it was important to share. Their exasperated responses of “*oh my god, yes”* and “*no one knows how hard this is*” affirmed its importance.

They agreed to share their experiences during COVID-19. That meeting was intense and moving. The youth shared deeply personal struggles and concerns–their own and those of their peers–as well as their hopes for how they could be supported more holistically. What follows is a summary of the challenges they have encountered during COVID-19 as queer and trans young people and their suggestions for how social workers and other professionals can support them. We share their quotes with their permission and approval from the University of Kansas Institutional Review Board. Although most youth elected not to be co-authors on this manuscript, their contributions are meaningful and essential to our understandings.

### “I feel so alone.”

Social and physical distancing is hard for everyone. Yet, the developmentally-situated drive for peer interaction coupled with budding independence from caregivers makes COVID-19 related physical distancing a unique hardship and challenge for adolescents. However, the Pride Alliance youth shared that for many queer and trans youth it also means hiding a significant part of themselves. Youth who have not “come out” to their parents or caregivers–or whose parents are aware but unsupportive–have lost access to spaces where they can be themselves and authentically express their sexuality or gender, such as school, community organizations, or simply with friends. Youth shared that some of their friends were afraid to come out due to fears of being kicked out of their home; a fear that exists regardless of, but is certainly intensified by, a global pandemic. They also discussed the challenge of being at home full-time with families who were unsupportive. As well as how difficult it was for them and their friends to have to interact with parents who were being openly anti-queer or anti-trans, with no opportunity to escape (even temporarily) from that environment.

### “I am having a really hard time finding time to connect …”

Independent of their own family situation, the youth expressed a shared sense of isolation. Even though they were not missing formal classes or schoolwork, they missed the safety and support they felt at school. School was often where they saw a social worker or counselor, could talk with a supportive teacher, or had friends with whom they could be their authentic selves. They shared how some of their friends had been questioning their sexuality or gender prior to the COVID-19 closures, and that they had accessed support from their friends and the school gender and sexuality alliance. Following school closures, these youth were engaging in the same demanding identity development work without the support they previously received. Thus, queer and trans youths’ sense of isolation was exacerbated by not just being physically alone, but by their inability to connect with supportive, affirming, and trusted mentors and friends.

### “Everyone at home is straight. I need queer people.”

Physical distancing not only kept youth from school supports and in challenging home situations full-time, but also kept them from other queer and trans youth. The youth shared how much they missed their queer community and connections. They found ways to interact with their local queer and trans friends using text, chat apps, and video chat, and appreciated being able to maintain connections in this way. However, they felt that their connections were not as strong as when they were in-person together. They talked about the importance of being around other queer people and how much they were missing that.

### “… gender dysphoria has gotten worse”

The Pride Alliance youth shared that the impacts of isolation, stigma at home, and a lack of community were exacerbating physical and mental health concerns among themselves and their friends. They described increased depression and anxiety, engaging in self-harming behaviors, and thoughts of suicide. They talked specifically about the harm of isolation for trans youth and how the inability to present authentically at home–via gender expression or in other ways–had resulted in increased gender dysphoria. One youth shared that they had been eating to cope, and that the weight gain was triggering dysphoria for them. Some youth also shared fears around the physical health of trans youth, particularly those who use chest binders to minimize the appearance of breasts, due to reduced lung capacity and COVID-19.

### “My parents check my phone …”

Given their increased isolation, lack of community, and intensified mental health difficulties the youth expressed significant concerns regarding the lack of formal and informal support available to them and their friends. They worried about friends they either had not heard from in a while, or who could not always be honest about their feelings over the phone or computer due to parental oversight. They shared how they or their friends could not talk with their therapist or counselor due to their parents listening in, and that even text/chat-based options were surveilled by parents. This was particularly worrisome for youth who were not out or who lived in unsupportive homes. This left them wondering *how* youth can access support while maintaining physical distancing, even in supportive homes.

## Implications and challenges for social work practice

As this essay was drafted, the social and health landscape of the COVID-19 pandemic has dramatically changed. We complete our writing as some states have begun to re-open libraries and other community spaces and are planning to reopen schools in Fall 2020. Still, COVID-19 numbers are increasing across the U.S. Restrictions that limit social contact will likely continue. Additionally, as the virus continues to surge and subside, there is no guarantee that any of these spaces will remain consistently open. When planning for this uncertain future, social workers should expect that there could be significant and ongoing impacts to social work practice for months (or even years) into the future. The Pride Alliance youth shared their hopes and suggestions for social workers and other practitioners for supporting queer and trans youth during these times.

### “Make us feel not so alone”

The youth discussed the importance of supportive adults reaching out, checking in, and connecting, but also identified significant barriers to these connections. For many, mental health professionals and resources were only available through school or community programs. Without access to those spaces, queer and trans youth identified a need for identity-specific mental health support. Tele-mentalhealth might be an option for queer and trans youth already working with a clinician before COVID-19, but even those youth may not have enough privacy at home to fully engage with their clinician–particularly on topics related to queer or trans identity and related stressors.

For youth with limited access to mental health professionals and privacy, it may be beneficial to consider text-based service. Text and app-based therapy have grown in popularity over the past decade as internet-enabled mobile devices have become more accessible. Emerging research demonstrates the effectiveness of text-based mental health support for isolated individuals, including aging individuals and people with disabilities. Clinicians, school social workers, and other direct-service providers should consider text-based outreach and support as an option for isolated queer and trans youth. It may be important for clinicians to provide opportunities for youth to indicate if they are able to be open and honest with the clinician in light of parental oversight, such as by providing both text- and audio-video-based options for youth to respond to questions and disclose information. It is also important for social workers to engage with parents to set boundaries around parental oversight that might adversely impact services for youth.

### “Validate us…this fucking sucks”

Relatedly, the youth shared that they appreciated the well-intentioned nature of social workers or other clinicians trying to help them “feel better” by “focusing on the positive” but suggested that they really just needed to be validated. They wanted social workers to hold space for them to express how bad it feels to be stuck at home and without community. Social and physical distancing does “suck” and hearing that from supportive adults made them feel affirmed. They agreed that the support did not need to end there–that they still needed resources and other types of support, but that a simple validation of “yes, you’re right, this sucks” can go a long way.

### “Ask us how we want help”

The youth made many recommendations for how practitioners could best serve queer and trans youth during COVID-19. Some were broad suggestions like those described above, but others were specific to their communities. For example, the member of the Pride Alliance who shared how weight gain during the pandemic had caused an increase in dysphoria indicated that they and their friends were in need of appropriately sized gender-affirming clothing. The youth suggested that practitioners should engage with queer and trans youth in their community to learn what kinds of help are wanted and needed. For the example above, a youth-centered practitioner could work with community partners and the local queer and trans community to put together a virtual or open-air queer clothing swap. Working collaboratively with queer and trans youth is essential to guide social workers in developing and providing services that are relevant and impactful.

In consultation and collaboration with queer and trans youth, organizations should work to develop accessible and affordable internet-based resources for youth, their parents, and teachers. In our research and practice we have seen a significant increase in demand for queer and trans-specific online programming since the onset of COVID-19, with community-based organizations scrambling to provide their programs via existing online platforms (e.g., Instagram, Discord). Such resources have at least begun to meet the increased demand for community connection, peer and professional support, and even just identity-affirming entertainment. However, an increased effort towards developing and evaluating intentional (instead of reactive) online programming is necessary to continue to effectively meet ongoing demands.

### “We need queer resources”

Finally, the youth discussed needing referrals to resources that were specific to queer and trans youth. They described how a general suicide hotline, for example, did not help if they needed to be sure they could talk about being trans with someone who is affirming. Additionally, they were not always in crisis, and instead just needed general queer and trans resources or support. Having access to and sharing these resources also validates youth’s queer or trans identity and demonstrates the social worker’s understanding of their unique needs. Social workers at every level should be knowledgeable about local and national online support groups, crisis hotlines, peer support, parent support, professional support, and e-therapy, as well as free digital access to books, magazines, and movies through schools, libraries, or other means. While a complete list of resources is beyond the scope of this essay, examples include Q Chat Space, the Trevor Project, and the Trans Lifeline. We also suggest that social workers connect with their local LGBTQ community organization(s).

## Conclusion

This reflexive essay highlights a point-in-time narrative of queer and trans youth navigating the complexities of school closures and physical/social distancing amidst the COVID-19 pandemic. Although it is the reflection of a small group of youth in one U.S. community, our practice and research suggest this is likely the experience of many queer and trans youth across the country. As COVID-19 impacts the ways in which social workers engage in practice, we must be mindful of the unique impacts on youth living in the complexities of marginalization without access to support and community. We all share the experience of navigating new realities under COVID-19. However, how we must navigate our new environment shifts dramatically when marginalization and oppression are fundamental to our realities.

